# Dosing variability in prescriptions of acetaminophen to children: comparisons between pediatricians, family physicians and otolaryngologists

**DOI:** 10.1186/1471-2431-13-64

**Published:** 2013-04-24

**Authors:** Yueh-Ching Chou, Shin-Yi Lin, Tzeng-Ji Chen, Shu-Chiung Chiang, Mei-Jy Jeng, Li-Fang Chou

**Affiliations:** 1 Department of Pharmacy, Taipei Veterans General Hospital, Taipei, Taiwan; 2 School of Pharmacy, Taipei Medical University, Taipei, Taiwan; 3 Institute of Pharmacology, School of Medicine, National Yang-Ming University, Taipei, Taiwan; 4 Institute of Hospital and Health Care Administration, School of Medicine, National Yang-Ming University, Taipei, Taiwan; 5 Section of Community Medicine, Department of Family Medicine, Taipei Veterans General Hospital, Taipei, Taiwan; 6 Institute of Emergency and Critical Care Medicine, School of Medicine, National Yang-Ming University, Taipei, Taiwan; 7 Department of Public Finance, National Chengchi University, Taipei, Taiwan; 8 Department of Pediatrics, Taipei Veterans General Hospital, Taipei, Taiwan

**Keywords:** Drug dosage calculations, Drug prescriptions, National health programs, Physician’s practice patterns

## Abstract

**Background:**

To estimate the extents of dosing variability in prescriptions of acetaminophen to children among pediatricians, family physicians and otolaryngologists.

**Methods:**

The acetaminophen prescriptions in the systematic sampling datasets from the National Health Insurance Research Database in Taiwan were analyzed. The distribution of dosages was measured and expressed in terms of coefficient of variation (CV). The analyses were stratified by patient’s age, prescriber’s specialty and preparation form.

**Results:**

From 13,868 prescribed items of acetaminophen in 2009, liquids accounted only for 11.1% (n = 1544). More than half (56.9%) of liquids were prescribed by pediatricians. The median dose (83.3 mg, n = 1683) of acetaminophen prescriptions in infants is around half of that in preschool children (166.7 mg, n = 3921), one-third in children (250.0 mg, n = 4926) and one-sixth in adolescents (500.0 mg, n = 3338). In infants, the prescriptions by pediatricians had the highest CV (86.7%), followed by family physicians (82.3%) and otolaryngologists (70.3%). The patterns were similar in preschool children and children, but the difference of CV among specialties narrowed down with the patient’s age.

**Conclusions:**

In acetaminophen prescriptions to children, pediatricians had a wider variability of dosages and a higher ratio of liquid preparations than family physicians and otolaryngologists. Further investigations can be undertaken to estimate the accuracy of dosing variability as an indicator of prescribing quality. Besides, child-suitable drug preparations should be promoted to ensure patient safety.

## Background

Children are not the exact miniatures of adults. The dosages of most medicines prescribed to children should be adjusted according to their ages and weights because children are not completely mature in development and the pharmacokinetics of a drug in them is hardly calculable [1]. For example, the half-life of acetaminophen, one of the most common antipyretics, is much slower in neonates than in adults [2]. Therefore, children are more vulnerable to dosing errors [3]. It has been also reported that not all physicians prescribed for children according to package inserts or guidelines [4-6]. Furthermore, the degree of prescribing discordance differed among specialties [7,8]. Most studies of cross-specialty prescription comparisons measured merely the appropriateness of indications [9], the choice of drugs [10,11], and the number of distinct drug items [12]. In some studies dealing with dosing problems, questionnaires [13], quizzes or chart reviews were usually adopted in research methods. To establish an indicator of prescribing quality in dosing within large-scale prescription databases, a more efficient approach is thus necessary.

In this study, we proposed a new method to estimate the extents of dosing variability in prescriptions to children among different specialties. Although prescription data were from a large nationwide health insurance claims database in Taiwan, the concept and procedures of our study could apply to studies of pediatric drug utilization in other countries.

## Methods

### Data source

The National Health Insurance (NHI) in Taiwan started in 1995 and covered 23,025,773 beneficiaries [14] (about 99% of 23,119,772 inhabitants [15]) at the end of 2009. Since 1999, the Bureau of National Health Insurance (BNHI) has released the claims data in electronic form to the National Health Research Institutes for research use under the project of National Health Insurance Research Database (NHIRD). Some dozen kinds of extracted datasets are available on application.

From the NHIRD, we obtained the ambulatory part of systematic sampling datasets in 2009. This kind of datasets was randomly sampled in a ratio of 1/500 from the entire datasets of the NHI in the year. It was composed of claims of 576,416 visits and 2,765,389 prescriptions in 2009. The data of each visit included the date of the consultation, the consulted physician’s specialty, and the patient’s age, sex and birthday. The data of each prescribed drug item included the drug identification number, dosage, frequency, and total amount. On the web site of the BNHI we could look up the ingredient, dosage form and strength of a drug according to its identification number. An interface for searching drug items according to the ingredient was also available.

### Subjects

This study has been approved by the institutional review board of Taipei Veterans General Hospital, Taipei, Taiwan.

This study is the cross-sectional, observational database analysis. We limited our analysis to prescriptions given to children not older than 18 years who were then grouped into infant (younger than 2 years), preschool child (2 to 5 years), child (6 to 11 years), and adolescent (12 to 17 years). To simplify the analysis, we further limited the analysis to drug items containing acetaminophen which was the most frequently prescribed ingredient as antipyretics and analgesics in children [16]. It was generally recommended that in patients younger than 13 years the dosage of acetaminophen should be adjusted according to body weight [17].

To calculate the dosage of acetaminophen in each prescription, we at first identified 288 drug items containing acetaminophen from the web site of the BNHI. The exact single dosage in unit of milligram was computed by multiplying the strength with the prescribed quantity, in either single-ingredient or compound preparations. In the analysis, the various drug forms were grouped into liquid (22 items, including syrup and elixir), tablet (200 items, including sugar-coated tablet and chewable tablet), capsule (62 items), and others (4 items, including oral granules and suppository). According to the reimbursement regulations of the NHI, liquid forms of drugs could only be prescribed to children under 13 years or on special situations. Furthermore, we analyzed only the regular prescriptions. The prescriptions as *statim*, *pro re nata* and *ut dictum* as well as outliers with dosages greater or less than 1.5 interquartile ranges were excluded from analysis.

To make a cross-specialty comparison of dosing variability, we chose only three major specialties: pediatrics, family medicine and otolaryngology. A recent study about child care within the NHI in Taiwan revealed that these three specialties covered 93.3% of ambulatory visits by children younger than 7 years [18]. Because people in Taiwan could freely choose physicians and healthcare settings without referral and the copayment was relatively low within the NHI, otolaryngologists became the second largest specialty in providing ambulatory health care, especially in treating diseases of the respiratory system that accounted for one third of ambulatory visits [19]. In Taiwan, the share of otolaryngology in ambulatory care could reach 9.5% in general population [19] and as high as 20.7% in children [18].

Although the NHI claims were lacking in the data of body weight, we supposed that the patients in each of three specialties have similar distributions of body weights. On this assumption, we would compare the distributions of acetaminophen dosages prescribed by physicians in three specialties. Our hypothesis was that pediatricians should be more likely to prescribe on the basis of a patient’s body weight. The dosing variability by pediatricians should be more marked than that by family physicians or otolaryngologists. The extent of difference in dosing variability between family physicians/otolaryngologists and pediatricians might serve as a quality indicator of prescribing.

### Statistical analysis

The database management software of Microsoft SQL 2008 (Microsoft Corp., Redmond, WA, USA) was used for data linkage and processing. At first, descriptive statistics were presented. Besides the mean and standard deviation (SD), we used the coefficient of variation (CV) to represent the dosing variability of acetaminophen prescriptions. The CV, defined as the ratio of the standard deviation to the mean, was thought to be more accurate than standard deviations to compare the variability [20] and usually applied to represent laboratory values, e.g. red blood cell distribution width in complete blood count. The results were stratified by child’s age group, prescriber’s specialty and dosage form of acetaminophen preparation. Then, the Kolmogorov-Smirnov test was used to explore whether the single dosages of acetaminophen prescriptions in each age group and prescriber’s specialty followed the normal distribution. The Wilcoxon rank-sum test was used to compare the distribution of acetaminophen dosages in family medicine or otolaryngology with that in pediatrics. A value of p < 0.05 was regarded as statistically significant. Statistical analyses were performed using the IBM SPSS statistical package, version 19.0.

## Results

From the sampling datasets of the NHIRD in 2009, we identified 110,825 visits by children. The visits to pediatricians, family physicians and otolaryngologists amounted to 73.1% (n = 80991) of all visits. Among the visits to these three specialties, acetaminophen was prescribed in 18,965 (23.4%) visits. After elimination of 5,193 prescriptions as *statim*, *pro re nata*, *ut dictum*, with outlier in dosage or obvious data entry error, 13,868 prescribed items of acetaminophen remained for further analysis: 1,683 to infants, 3,921 to preschool children, 4,926 to children, and 3,338 to adolescents (Table 1). Pediatricians accounted for 38.6% (n = 5,353) of acetaminophen prescriptions, family physicians for 30.8% (n = 4,276) and otolaryngologists for 30.6% (n = 4,239). The share of pediatricians decreased with the patient’s age: from 57.2% in infants to 24.1% in adolescents.

**Table 1:** Features of patients receiving prescriptions (N = 13868) of acetaminophen

	**Infant**	**Preschool child**	**Child**	**Adolescent**
	**(<2 y/o)**	**(2–5 y/o)**	**(6–11 y/o)**	**(12–17 y/o)**
No. of patients	1683	3921	4926	3338
Male (%)	763 (45.3)	1838 (46.9)	2345 (47.6)	1578 (47.3)
Female (%)	919 (54.6)	2083 (53.1)	2580 (52.4)	1760 (52.7)
Missing (%)	1 (0.1)		1 (0.0)	
Patient’s age, mean ± SD, y	1.2 ± 0.5	4.0 ± 1.1	8.8 ± 1.7	14.8 ± 1.6
Prescriber’s specialty				
Pediatrics (%)	962 (57.2)	1752 (44.7)	1833 (37.2)	806 (24.1)
Family medicine (%)	374 (22.2)	1007 (25.7)	1528 (31.0)	1367 (41.0)
Otolaryngology (%)	347 (20.6)	1162 (29.6)	1565 (31.7)	1165 (34.9)

As to drug forms, 1,544 (11.1%) prescribed items belonged to liquids, 12,112 (87.3%) to tablets, 205 (1.5%) to capsules, and 7 (0.1%) to others. While liquids accounted for 32.1% of acetaminophen prescriptions in infants, the share decreased to 17.6% in preschool children and 6.4% in children. Pediatricians were more likely to prescribe liquid forms of acetaminophen than family physicians and otolaryngologists. But the cross-specialty disparity diminished with the patient’s age (Table 2).

**Table 2:** Forms of acetaminophen preparations in 13868 prescriptions, stratified by patient’s group and prescriber’s specialty

	**No. of prescriptions**	**Liquid**^**a **^**(%)**	**Tablet (%)**	**Capsule (%)**	**Others**^**b **^**(%)**
Infant					
Pediatrics	962	358 (37.2)	594 (61.7)	10 (1.0)	
Family medicine	374	109 (29.1)	257 (68.7)	8 (2.1)	
Otolaryngology	347	73 (21.0)	270 (77.8)	2 (0.6)	
Preschool child					
Pediatrics	1752	378 (21.6)	1352 (77.2)	22 (1.3)	2 (0.6)
Family medicine	1007	169 (16.8)	797 (79.1)	41 (4.1)	
Otolaryngology	1162	143 (12.3)	1010 (86.9)	4 (0.3)	
Child					
Pediatrics	1833	142 (7.7)	1649 (90.0)	42 (2.3)	5 (0.4)
Family medicine	1528	100 (6.5)	1388 (90.8)	40 (2.6)	
Otolaryngology	1565	72 (4.6)	1458 (93.2)	35 (2.2)	
Adolescent					
Pediatrics	806		806 (100.0)		
Family medicine	1367		1366 (100.0)	1 (0.1)	
Otolaryngology	1165		1165 (100.0)		

^a^ including syrup and elixir.

^b^ including suppository and oral granules.

**Figure 1:**
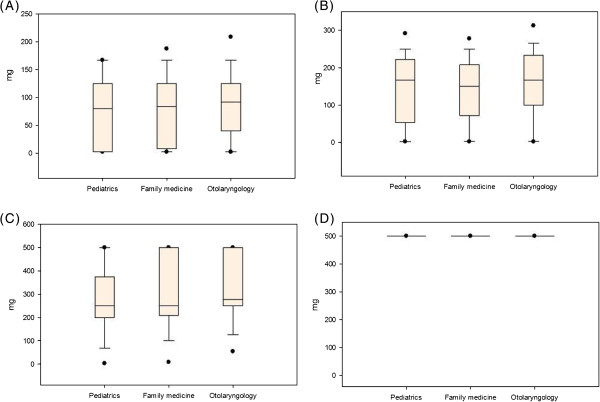
**Distribution of dosages in acetaminophen prescriptions in each patient’s group, stratified by prescriber’s specialty: (A) infant; (B) preschool child; (C) child; and (D) adolescent.** Box plots show the median and interquartile ranges of acetaminophen dosages. The “whiskers” outside the box indicate the 10th and 90th percentiles; solid circles indicate the 5th and 95th percentiles.

About the distribution of acetaminophen dosage, the median dose (83.3 mg) of acetaminophen prescriptions in infants is around half of that in preschool children (166.7 mg), one-third in children (250.0 mg) and one-sixth in adolescents (500.0 mg) (Figure 1). In average, otolaryngologists prescribed the highest dosage in infants, preschool children and children, while family physician prescribed a higher dosage than pediatricians only in infants and children (Table 3). The cross-specialty disparity of dosage disappeared in adolescents.

**Table 3:** Distribution of acetaminophen dosages in 13868 prescriptions, stratified by patient’s group and prescriber’s specialty

	**No. of prescriptions**	**Median, mg**	**Mean (SD), mg**	**Coefficient of variation,%**	**P value**^**a**^
Infant					
Pediatrics	962	80.0	72.3 (62.7)	86.7	-
Family medicine	374	83.3	79.6 (65.5)	82.3	0.033*
Otolaryngology	347	91.7	91.0 (64.0)	70.3	<0.001*
Preschool child					
Pediatrics	1752	166.7	145.8 (98.8)	67.8	-
Family medicine	1007	150.0	140.3 (90.1)	64.2	0.135
Otolaryngology	1162	166.7	162.3 (92.1)	56.7	<0.001*
Child					
Pediatrics	1833	250.0	279.1 (148.2)	53.1	-
Family medicine	1528	250.0	295.1 (150.0)	50.8	0.007*
Otolaryngology	1565	277.8	305.4 (140.7)	46.1	<0.001*
Adolescent					
Pediatrics	806	500.0	498.1 (13.9)	2.8	-
Family medicine	1367	500.0	498.1 (14.0)	2.8	0.747
Otolaryngology	1165	500.0	497.2 (17.2)	3.5	0.201

^a^ Wilcoxon rank-sum test.

^*^ statistically significant.

As to measurement of dosing variability in terms of CV, the acetaminophen prescriptions in adolescents had the lowest CV (2.8-3.5%). In infants, the prescriptions by pediatricians had the highest CV (86.7%), followed by family physicians and otolaryngologists. The patterns were similar in preschool children and children, but the difference of CV among specialties narrowed down with the patient’s age (Table 3). Within the same specialty, CV also decreased with the patient’s age.

## Discussion

Our results revealed that the relative ratios of median doses of acetaminophen among all age groups were quite similar to those of median body weights in CDC Growth Charts [21] and to those of recommended doses in pharmacopeia [22]. It implied that most physicians paid attention to the child’s body weight or age in prescribing. Thus, even though the information of body weight was not available in the claims datasets, we might infer from the dosing variability among specialty groups that the prescribing quality of physicians with narrower variability needed to be further scrutinized.

On the other hand, we could neither directly prove that the CV of dosages was suitable to serve as a measurement of prescribing quality. According to the Child Growth Standards published by the World Health Organization [23], the variation of body weight in infants is most marked and the extent of variation declines with age. In our study, the CV in each specialty also decreased with children’s age. Besides, children aged 12 years and older can have the same dosage (500 mg) of acetaminophen as adults, without regard to body weight [17]. In our study, the CV in each specialty was also negligibly small in adolescents (Table 3). The pattern of CVs reflected their usefulness in cross-specialty comparisons.

In our study, otolaryngologists had the narrowest dosing variability in children younger than 12 years. It was understandable that otolaryngologists with the background of surgically-oriented training might not be familiar with pediatric prescription or accustomed to dosing practice. The discrepancy of practice patterns between otolaryngologists and pediatricians had been reported, especially in treatment of acute otitis media and tonsillitis [24,25]. A survey of otolaryngologists and pediatricians about deficiency of cross-training in Canada had also revealed that otolaryngologists felt a need of medication and dosage knowledge to be taught by pediatricians during otolaryngology residency [26]. Modification of training programs or continuing education might be considered to resolve the cross-specialty discrepancy.

As hypothesized in our study, the prescriptions by pediatricians had indeed higher CVs of acetaminophen dosages than those by family physicians and otolaryngologists. The cross-specialty differences of CVs decreased with patient’s age and disappeared in adolescents. Our study also revealed that pediatricians prescribed much more preparations in liquid forms than family physicians and otolaryngologists did. In Taiwan, many physician clinics still have dispensing practice [27,28]. The formularies available to family physicians and otolaryngologists might lack preparations suitable for child use. Dose adjustment in tablets and capsules was less easily performed than in liquid preparations. Beside physician’s prescribing skills, the problem of formulary might be another explanatory factor of cross-specialty discrepancy in dosing variability. Because the sample size in each subgroup stratified by age and preparation form would become too small, we could not ascertain the role of preparation form in our study. However, our findings still highlighted the need of provision of child-suitable preparations as currently promoted by major global health organizations [29,30].

Our study with claims data from NHIRD had some limitations. First, the indications for acetaminophen prescribing were not analyzed in our study. The claims record of an ambulatory visit contained three diagnostic fields. It was technically difficult to attribute acetaminophen to a diagnosis. Besides, acetaminophen was usually used to alleviate symptoms rather than to cure diseases. Second, for children aged between one and 12 years, the body weight increased almost linearly with age. Because of sample sizes, we adopted broader grouping of patients instead of presenting the results with a resemblance to growth charts. However, for children aged under one year, especially neonates, pediatricians might be the main health providers, potentially contributing to a higher dosing variability of acetaminophen prescriptions in infants treated by pediatricians. Third, from the prescription records we could understand whether acetaminophen was taken in a divided tablet or capsule. In real life, splitting or grinding might lead to more dosage deviation than physician’s prescribing did. This issue would go beyond the scope of our study.

## Conclusions

In acetaminophen prescriptions to children, pediatricians had a wider variability of dosages and a higher ratio of liquid preparations than family physicians and otolaryngologists. Further investigations can be undertaken to estimate the accuracy of dosing variability as an indicator of prescribing quality. Besides, child-suitable drug preparations should be promoted to ensure patient safety.

## Abbreviations

BNHI: Bureau of National Health Insurance; CV: Coefficient of variation; NHI: National Health Insurance; NHIRD: National Health Insurance Research Database; SD: Standard deviation.

## Competing interests

The authors declared that they have no competing interests.

## Authors’ contributions

YCC, SYL, MJJ and TJC involved in the study design. YCC, SYL, SCC, LFC and TJC analysis and interpretation of the data. SYL wrote the first draft of the manuscript. TJC and YCC are revising it critically for important intellectual content. All authors read and approved the final manuscript.

## Pre-publication history

The pre-publication history for this paper can be accessed here:

http://www.biomedcentral.com/1471-2431/13/64/prepub
